# Higher BMI is linked to an increased risk of heart attacks in European adults: a Mendelian randomisation study

**DOI:** 10.1186/s12872-020-01542-w

**Published:** 2020-05-29

**Authors:** Benjamin Adams, Lauren Jacocks, Hui Guo

**Affiliations:** Centre for Biostatistics, Division of Population Health, Health Services Research and Primary Care, Faculty of Medicine, Biology and Health, The University of Manchester, Manchester, UK

**Keywords:** BMI, Heart attacks, Causal relationship, Mendelian randomisation

## Abstract

**Background:**

BMI has been implicated as a risk factor for heart disease as a whole in multiple studies. Heart attack is one of the common complications of this disease. The aim of this study is to explore if elevated level of BMI causes an increase in the risk of heart attacks.

**Methods:**

We used two Mendelian randomisation (MR) methods: inverse variance weighted estimation and robust adjusted profile score (RAPS) on the basis of summary data of adulthood BMI from Genetic Investigation of Anthropometric Traits consortium and heart attack data from the UK Biobank. BMI associated single nucleotide polymorphisms (SNPs) were used as instrumental variables.

**Results:**

Seventy-two independent SNPs were associated with BMI (*P* < 5 × 10^− 8^). Using these SNPs as instruments, BMI was found to be causally associated with heart attacks in inverse variance weighted MR analysis. The risk of heart attacks increased by 0.8% per 1-SD (or 4.5 kg/m^2^) increase in BMI (OR = 1.008 with 95% CI (1.003, 1.012), *P* = 0.001). RAPS provided concordant results (OR = 1.007 with 95% CI (1.002, 1.012), *P* = 0.004).

**Conclusions:**

This current study is the first to use MR to investigate causal relationship between BMI and heart attacks. Our findings suggest that high level of BMI may cause increased risk of heart attacks.

## Background

### The obesity pandemic

It is no secret that being overweight is increasingly becoming the norm, with the latest research briefing in the houses of parliament claiming that 28.7% of adults in the UK are obese and a further 35.6% are overweight [1]. Obesity measured as a BMI > 30 kg/m^2^ has been linked to a plethora of ailments through observational studies [2–6]. According to the Office for National Statistics, in 2018, the leading cause of deaths in the UK was ischaemic heart disease for men, accounting for 13.2% of deaths [7].

BMI has been implicated as a risk factor for heart disease as a whole [8]. Many studies, however, only seem to tackle more generalised terms of the disease. A previous observational study has identified a link between heart attacks and BMI [4]. They observed a cohort of 899 obese individuals in adults between 35 and 74. After 10 years of observations, the study concluded that obesity was not an independent cardiovascular risk factor. This study is statistically underpowered due to small sample size. Furthermore, like other observational studies, an obvious issue is that it may suffer from problems of confounding (e.g. smoking, alcohol abuse) [9] and other sources of bias [10]. Conclusions from these studies have limited use as clinical development of treatments requires well-targeted causal factors [11–13]. In our study, we can put this statement to the test as we employ the use of Mendelian randomisation (MR) to bypass these issues, with the aim to better infer causality of obesity on heart attacks in a population of white European individuals.

## Methods

### Study design

In our investigation, we have applied two-sample MR to explore if an elevated level of BMI (regarded as exposure) causes increased risk of heart attacks (regarded as outcome) using BMI associated single nucleotide polymorphisms (SNPs) as the instruments.

Two-sample MR requires participants from two separate studies – one for exposure and the other for outcome, where the individuals do not overlap since overlapping data sets would lead to our results suffering from inflated type 1 error rates [14]. However, the two samples must be representative of the same population. Thus, we have used summary data of European descendants from two independent studies: Genetic Investigation of Anthropometric Traits (GIANT) consortium (for BMI) [15] and the UK Biobank (for heart attacks) [16].

### BMI data - GIANT

The genetic instruments were SNPs selected from GIANT [15]. This study consists of a meta-analysis of a population from European ancestry containing 322,154 individuals from 114 studies. Summary level data was extracted (see Summary Data section for more details) for BMI associated SNPs (*P* < 5 × 10^− 8^). These SNPs were further filtered by clumping carried out using the MR-Base platform [17] to ensure that the final set of instruments in MR analysis were independent of one another. Essentially the SNPs in linkage disequilibrium (LD) (*R*^*2*^ ≥ 0.001) were clumped together with only the SNP with the lowest *p*-value being retained.

### Heart attack data - UK biobank

The UK Biobank data contains approximately half a million individuals aged between 40 and 69 years [18]. The participants were recruited from across the UK between 2006 and 2010 and asked to provide information via questionnaires, interviews, anthropometric measures and samples (e.g. blood, urine and saliva). The summary data we used for heart attacks was from GWAS results by the Neale lab who carried out rigorous quality control (QC) checks [16]. These checks whittled down the individuals involved to only QC positives (*n* = 337,199). The filter which caused the largest reduction in participants was the restriction to white British genetic ancestry only. Participants were also removed if they were closely related to other individuals in the study or had sex chromosome abnormalities. To learn more about the QC process please see the Neale Lab website [16].

As of 2018, over 92 million autosomal SNPs (directly genotyped or imputed) were available for analysis. All these SNPs were further restricted by minor allele frequency (MAF) > 0.1%, Hardy-Weinberg Equilibrium (HWE) *p*-value > 1 × 10^− 10^ in the QC positive individuals and an imputation score INFO > 0.8 leaving approximately 13.8 million SNPs for analysis [16].

For the heart attack data obtained from the UK Biobank, participants were asked in a survey on their medical history to categorically state if they had had a doctor diagnosed heart attack or stroke, or suffered from angina or high blood pressure [19]. Participants could also state if they had suffered from none of the above. The data from this survey was converted into binary (1: suffered from a doctor diagnosed heart attack, 0 otherwise).

### Summary data

Instead of using individual-level data, one of the advantages of MR analysis is leverage summary statistics at the SNP level (estimated SNP effects, standard errors and corresponding *p*-values from regression models, effect alleles and other alleles along with their frequencies). These summary statistics from many large-scale genome wide association studies (GWAS) are now made publicly available.

Summary data of BMI and heart attacks were extracted separately, for BMI associated SNPs, from the GIANT and UK Biobank studies [15, 16]. In GIANT, data were standardised such that per unit change in BMI corresponds to 1 standard deviation (or 4.5 kg/m^2^) change in BMI. We then carried out harmonisation using the TwoSample MR package in R to make sure that the effects of a SNP on the outcome and exposure were relative to the same allele, which produced one merged dataset for our MR analysis.

### Statistical analysis

**Fig. 1:**
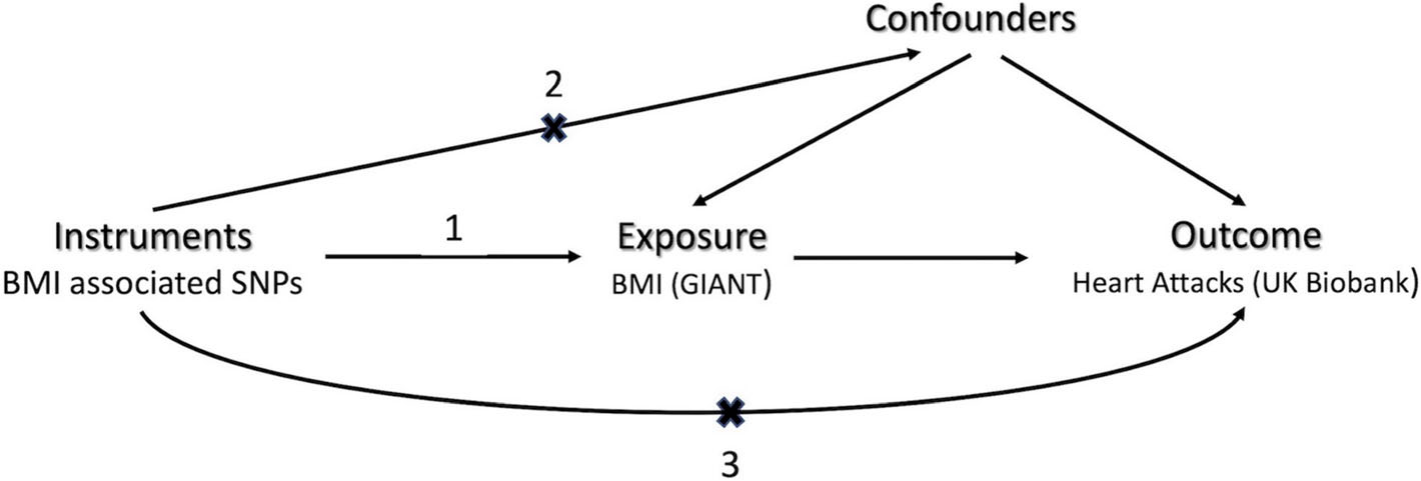
Schematic representation of assumptions of our MR analysis. In this investigation, MR has been used to test the causal relationship between BMI and heart attacks in adulthood. The numbers indicate the three assumptions: 1) instrumental SNPs are associated with BMI; 2) instrumental SNPs are not associated with confounders; 3) instrumental SNPs do not have direct effects on the risk of heart attacks, i.e., their effects on heart attacks are mediated only through BMI

Before the advent of MR, observational studies were greatly limited by problems of unobserved confounding. These limitations rendered many findings lack of causal interpretations. MR, however, circumvents these difficulties by mimicking randomised controlled experiment and assuming that the instruments (SNPs) fulfil three criteria listed in Fig. 1.

We used two MR methods: inverse variance weighted (IVW) estimation and MR robust adjusted profile score (RAPS) to estimate causal effect of BMI on heart attacks (log odds ratio), its standard error and corresponding *p*-value [20, 21]. Both of the methods require that instrumental SNPs are associated with the exposure and mutually independent. In IVW estimation, causal effect of an exposure on an outcome is first estimated as the ratio of the SNP-outcome association estimate to the SNP-exposure association estimate for each instrumental SNP. The IVW estimate is weighted average of these estimated causal effects, where each of the estimates is weighted by the inverse of their variance [22]. An obvious advantage of IVW is its use of multiple instruments to improve statistical power. However, it is prone to bias due to weak instruments. RAPS enhances the IVW estimation by replacing each weight with a function form of the causal effect and the variation of the instrument strength [23]. This approach provides more robust results in the presence of weak instruments than IVW estimate.

## Results

### Higher BMI causes increased risk of heart attacks in European descendants

**Fig. 2:**
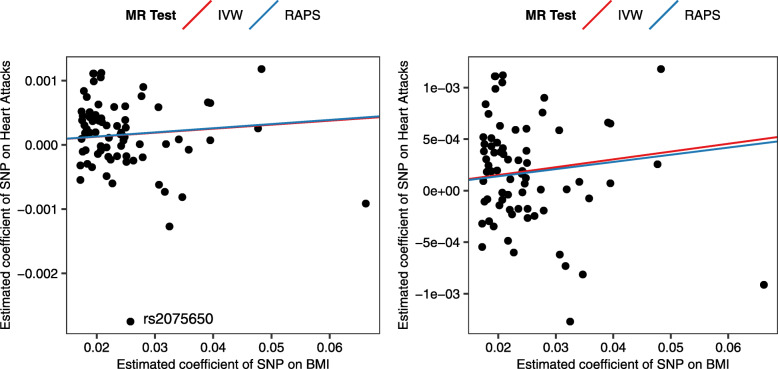
MR results with (left panel) and without (right panel) outlier SNP rs2075650. Each dot, corresponding to one of the instrumental SNPs of the investigation, represents estimated regression coefficient of the SNP on BMI (horizontal axis) and on heart attacks (vertical axis). The slopes are causal effects (log odds ratios) of BMI on heart attacks estimated from two MR methods: inverse variance weighted (IVW) estimation (red) and robust adjusted profile score (RAPS) (blue)

Seventy-three independent SNPs that are at least 10,000 kb apart were found associated with BMI (Table 1). In our MR analysis we identified an outlier. The estimated causal effect of BMI on heart attacks obtained based on this SNP was more than 3 standard deviations away from the average causal effect calculated from all the 73 SNPs. We researched that particular outlier rs2075650 (located at bottom left, left panel of Fig. 2) in order to determine if it exhibits pleiotropy – it also influences other health conditions apart from heart attacks. According to the Ensembl database, this SNP is located in the *TOMM40* gene and in close proximity to the *APOE* gene but may also influence Alzheimer’s disease and age-related macular degeneration [24].

**Table 1:** Summary results of BMI associated independent instrumental SNPs

SNP	Chr	Position	EA	OA	EAF	Beta	SE	P	Gene
rs1343424	1	49,376,820	C	T	0.251	0.02	0.004	8.56E-09	*AGBL4*
rs2422134	1	72,514,701	C	G	0.508	−0.022	0.003	2.49E-13	*NEGR1*
rs6690871	1	74,977,277	G	A	0.407	0.022	0.003	1.28E-12	*LRRC53*
rs505066	1	96,882,671	A	C	0.285	0.025	0.004	2.35E-09	*(UBE2WP1)*
rs7550711	1	110,082,886	T	C	0.026	0.066	0.009	9.51E-14	*GPR61*
rs4478775	1	177,770,097	A	G	0.223	0.025	0.004	1.98E-11	*SEC16B*
rs2820292	1	201,784,287	A	C	0.432	−0.02	0.003	1.58E-10	*NAV1*
rs13411762	2	554,109	C	T	0.928	0.033	0.006	5.93E-09	*(TMEM18)*
rs13391869	2	606,213	G	A	0.848	−0.022	0.004	1.65E-08	*(TMEM18)*
rs10495749	2	24,730,847	A	G	0.231	−0.021	0.004	3.21E-09	*NCOA1*
rs11126666	2	26,928,811	G	A	0.745	−0.021	0.003	5.71E-10	*KCNK3*
rs13011109	2	58,857,419	G	C	0.612	0.018	0.003	6.84E-09	*FANCL*
rs6545714	2	59,307,725	G	A	0.399	0.018	0.003	3.19E-09	*(FANCL)*
rs2890652	2	142,959,931	T	C	0.839	−0.028	0.005	2.24E-08	*(LRP1B)*
rs1528435	2	181,550,962	T	C	0.62	0.018	0.003	4.68E-09	*(UBE2E3)*
rs7599312	2	213,413,231	G	A	0.732	0.022	0.003	4.88E-11	*(ERBB4)*
rs10510554	3	25,099,776	T	C	0.431	−0.018	0.003	1.78E-09	*RARB*
rs1916801	3	61,187,046	A	T	0.595	0.019	0.003	8.13E-10	*FHIT*
rs3849570	3	81,792,112	A	C	0.348	0.019	0.003	1.61E-08	*GBE1*
rs10511073	3	85,653,460	G	A	0.646	−0.021	0.003	1.46E-10	*CADM2*
rs16851483	3	141,275,436	G	T	0.934	−0.048	0.008	1.77E-10	*RASA2*
rs4494964	3	185,776,491	T	C	0.142	−0.04	0.005	1.39E-15	*ETV5*
rs13144044	4	45,082,236	C	G	0.534	−0.025	0.004	1.02E-11	*(GNPDA2)*
rs17001654	4	77,129,568	C	G	0.852	−0.031	0.005	3.88E-09	*SCARB2*
rs13107325	4	103,188,709	C	T	0.925	−0.048	0.007	1.15E-12	*SLC39A8*
rs11727676	4	145,659,064	C	T	0.096	−0.036	0.006	1.11E-08	*HHIP*
rs6871667	5	74,604,742	A	G	0.401	−0.017	0.003	1.44E-08	*(HMGCR)*
rs280275	6	50,668,612	A	G	0.952	−0.039	0.007	4.46E-09	*(TFAP2D)*
rs4141973	6	50,946,521	T	C	0.134	0.028	0.005	5.51E-09	*(FTH1P5)*
rs9400239	6	108,977,663	C	T	0.706	0.019	0.003	6.10E-09	*FOXO3*
rs13191362	6	163,033,350	A	G	0.875	0.028	0.005	3.94E-09	*PARK2*
rs1167827	7	75,163,169	A	G	0.435	−0.02	0.003	4.64E-10	*HIP1*
rs2245368	7	76,608,143	T	C	0.833	−0.032	0.006	1.34E-08	*DTX2P1*
rs2922763	8	76,573,711	G	T	0.274	−0.024	0.004	2.73E-10	*(HNF4G)*
rs2033732	8	85,079,709	C	T	0.744	0.019	0.004	2.06E-08	*(RALYL)*
rs4740619	9	15,634,326	T	C	0.551	0.018	0.003	3.87E-09	*CCDC171*
rs1412234	9	28,410,683	C	T	0.33	0.024	0.004	4.97E-09	*LINGO2*
rs6477694	9	111,932,342	C	T	0.354	0.017	0.003	9.95E-09	*(FRRS1L)*
rs1928295	9	120,378,483	C	T	0.43	−0.019	0.003	6.62E-10	*(TLR4)*
rs10733682	9	129,460,914	A	G	0.473	0.017	0.003	9.95E-09	*LMX1B*
rs7899106	10	87,410,904	A	G	0.95	−0.04	0.007	1.32E-08	*GRID1*
rs17094222	10	102,395,440	C	T	0.213	0.025	0.004	2.83E-11	*(PAX2)*
rs17747324	10	114,752,503	C	T	0.227	−0.023	0.004	1.16E-09	*TCF7L2*
rs1374262	11	8,458,749	G	A	0.532	0.017	0.003	1.44E-08	*STK33*
rs4514364	11	27,456,059	C	T	0.316	0.02	0.003	1.72E-09	*LGR4*
rs2176598	11	43,864,278	T	C	0.246	0.02	0.004	1.90E-08	*HSD17B12*
rs2856650	11	47,365,199	G	A	0.703	−0.02	0.003	9.87E-10	*MYBPC3*
rs12286929	11	115,022,404	G	A	0.527	0.022	0.003	1.28E-12	*(CADM1)*
rs2720296	12	50,169,070	G	A	0.613	−0.02	0.003	9.87E-10	*(LSM6P2)*
rs11057405	12	122,781,897	A	G	0.106	−0.031	0.006	1.19E-08	*CLIP1*
rs7988412	13	28,000,282	C	T	0.815	−0.026	0.005	1.10E-08	*GTF3A*
rs2797084	13	54,071,824	T	C	0.86	− 0.032	0.006	6.12E-09	*(OLFM4)*
rs10132280	14	25,928,179	A	C	0.3	−0.023	0.003	6.68E-12	*(STXBP6)*
rs2370982	14	79,890,677	T	C	0.216	0.025	0.005	2.43E-08	*NRXN3*
rs3736485	15	51,748,610	A	G	0.461	0.018	0.003	6.84E-09	*DMXL2*
rs12905371	15	67,845,930	C	T	0.736	0.021	0.004	4.46E-09	*MAP 2 K5*
rs12448257	16	3,599,655	G	A	0.784	−0.024	0.004	8.42E-10	*NLRC3*
rs2531995	16	4,013,467	C	T	0.379	−0.024	0.004	8.46E-10	*ADCY9*
rs2354584	16	19,778,575	A	G	0.87	0.035	0.006	1.10E-09	*IQCK*
rs2650492	16	28,333,411	A	G	0.298	0.021	0.004	1.67E-09	*SBK1*
rs2303222	16	31,085,470	C	T	0.378	−0.018	0.003	1.78E-09	*ZNF668*
rs1477199	16	53,712,135	A	G	0.864	−0.024	0.004	1.90E-08	*RPGRIP1L*
rs7205986	16	53,755,146	G	A	0.511	0.025	0.003	3.68E-16	*FTO*
rs3026101	17	5,280,440	C	T	0.301	0.018	0.003	1.23E-08	*NUP88*
rs12949279	17	78,558,411	C	T	0.439	−0.018	0.003	8.25E-09	*RPTOR*
rs891389	18	21,087,531	C	T	0.655	−0.021	0.004	2.20E-08	*RMC1*
rs7243357	18	56,883,319	G	T	0.176	−0.022	0.004	2.90E-08	*(GRP)*
rs4940929	18	57,803,890	G	C	0.583	0.027	0.004	3.43E-11	*(MC4R)*
rs9944545	18	57,958,244	T	C	0.297	0.034	0.003	5.66E-24	*(MC4R)*
rs17724992	19	18,454,825	A	G	0.733	0.019	0.004	1.49E-08	*PGPEP1*
rs29944	19	34,306,898	A	G	0.327	−0.018	0.003	2.07E-08	*(KCTD15)*
*rs2075650*	*19*	*45,395,619*	*A*	*G*	*0.854*	*0.026*	*0.005*	*4.92E-09*	*TOMM40*
rs9304665	19	47,602,577	A	T	0.765	0.025	0.004	7.61E-09	*ZC3H4*

Table 1 lists summary results of the 73 independent SNPs used as instruments in our MR analysis. These SNPs were associated with BMI (*P* < 5 × 10^−8^) in the European adults in the GIANT study. One SNP rs2075650 appeared to be an outlier. We re-did MR analysis without this SNP. SNP: single nucleotide polymorphism, Chr: chromosome, Position: location of the SNP in the chromosome according to Build 37, EA: effect allele, OA: the other allele, EAF: effect allele frequency. Beta, SE and P are estimated SNP effect, standard error and *p*-value from the GIANT GWAS study. Gene: genes of the SNPs located in or close to. For intergenic SNPs, their nearest genes are provided in brackets

Both *TOMM40* and *APOE* are located at 19q13.32 in the genome in chromosome 19 [25]. The protein encoded by *TOMM40* is a channel-forming subunit of the translocase of the mitochondrial outer membrane. It is essential for the import of protein precursors into the mitochondria. The protein APOE, coding for apolipoprotein E, binds to fats in the blood to form lipoproteins. This allows for the transport of fats throughout the bloodstream, preventing the build-up of fats in the blood that could otherwise contribute to heart disease phenotypes if left there. Both of the genes have been linked to white matter integrity in the brain and the onset of Alzheimer’s disease, although nothing has been conclusively proven [26].

We re-ran MR by excluding the outlier rs2075650 as a sensitivity analysis. Although this SNP might introduce pleiotropy, our MR results are concordant, with or without rs2075650, as shown in the two panels of Fig. 2. The slopes of the lines represent estimated log odds ratios. Both of the MR methods (IVW in red, RAPS in blue) have shown evidence for a positive causal effect of BMI on heart attacks. For simplicity, we only report estimated causal effect (odds ratio) from MR using the remaining 72 instruments in Table 2.

**Table 2:** Estimated causal effect of BMI on heart attacks from two MR methods

MR Method	Estimated OR	95% CI	*P*-value
IVW	1.008	(1.003, 1.012)	0.001
RAPS	1.007	(1.002, 1.012)	0.004

Table 2 lists results from the two MR methods: inverse variance weighted (IVW) estimation and robust adjusted profile score (RAPS) using the 72 independent BMI-associated SNPs as instruments. Estimated OR: estimated odds ratio. It states the estimated change in the risk of having a heart attack, for every 4.5 kg increase of weight per square metre. 95% CI: 95% confidence interval.

The risk of heart attacks increased by 0.8% (in IVW) and 0.7% (in RAPS) per 1-SD (or 4.5 kg/m^2^) increase in BMI. The 95% CIs from both of the methods do not include value 1 implying that the estimated positive causal effect of BMI on the risk of heart attacks is unlikely to have occurred due to chance alone.

## Discussion

### Surrounding research

Our two-sample MR analyses showed evidence for a positive causal effect of BMI on the risk of heart attacks. There are many previous studies that have used MR and reported a causal link between BMI and cardiovascular diseases [3, 27, 28]. To the best of our knowledge, however, this current study is the first to use MR to explore if increased BMI causes an increase specifically in the risk of heart attacks. This is important as the root causes of cardiovascular diseases as a whole are generally too broad to find the biological reasoning behind the mechanisms. Choosing a smaller target makes interpretation of these mechanisms much clearer.

This new evidence aids the growing theory that obesity is associated with a heightened risk of a plethora of cardiac related disease phenotypes. Such studies that have also contributed to this theory include one which linked BMI to heart failure using the ENGAGE consortium (*n* = 19,384) [29], and another which used the UK Biobank to link BMI to aortic valve stenosis (*n* = 367,703) [27]. The latter study also related BMI to a lot of other cardiovascular diseases and so came the closest to detailing the causal effect of BMI on heart attacks although they did not explicitly state this. Furthermore, the BMI data they used was sourced elsewhere to GIANT. Overall, it seems that much of existing research does support similar evidence that BMI is related to a large array of heart disease, including heart attacks.

### Biological mechanism

High BMI values have been found to cause hypertension through MR tests [30, 31]. These results were reported to be independent of age, sex or confounders such as smoking and alcohol intake. Hypertension adds force onto the arterial walls as blood flows through. Continued exposure to these larger forces increases the chances of arterial wall damage and the accumulation of cholesterol, other lipids and therefore plaques. Plaques can narrow the diameter of the arteries that provide oxygenated blood to the heart muscle. This is usually asymptomatic but cases of angina or other chest pains are common, especially when one is exerting themselves. Eventually, the plaque will either grow to fully occlude the lumen of the artery or potentially break off to block a narrower section downstream of the plaque. In both instances, blood flow is cut off to the heart and so the individual experiences a heart attack (myocardial infarction) which can be fatal.

Elevated BMI has also been associated with increased blood interleukin 6 (IL-6) and expression of its receptor IL-6R [6]. IL-6 has been linked to the development of atherosclerotic plaques through increasing the rate of synthesis of its components. Lipid concentrations, blood glucose, C-reactive protein (CRP), interleukin 18 and adiponectin are also commonly used as markers for heart attacks and have been shown to be increased in the obese [32].

Though this is not an exhaustive list of the potential biological mechanisms behind the causal association between obesity and heart attacks, they are some of the most common causes in the UK population. It is important to note that just because this association is found through genetic instruments, it does not mean that lifestyle changes cannot alter the chances of a heart attack outcome. Regular exercise and a low-fat diet, along with weight loss have been shown to reduce the chances of heart attacks [32].

### Strengths & Limitations

Using UK Biobank and GIANT was advantageous for our investigation due to their large sample size and high quality of data. Focusing only on European ancestry and imputed SNPs with high accuracy mitigated primary confounding factors e.g. population structure and imputation errors.

MR is the star attraction of this investigation. It has allowed us to investigate the causal association of BMI on heart attacks without the usual pitfalls of confounders being an issue. The MR design has many strengths as it relies solely on genetic instruments. Alleles are randomly sorted and become fixed at conception and MR then directly tests these alleles, which bypasses biases due to confounding and reverse causality.

A limitation very common to MR studies is pleiotropy, and this study is no exception. Pleiotropy was the reason for the removal of SNP rs2075650 due to the belief that the SNP was also associated with Alzheimer’s disease. The use of a robust method RAPS means that our analysis was improved by reducing the likelihood of biased results due to weak instruments.

A limitation of our study was the terminology used in the UK Biobank data with reference to the outcome. The term ‘heart attack’ can be open to interpretation when it comes to what actually went wrong in the body. Not only because there are five recognised forms of heart attacks [33], but also because to a member of the public answering the UK Biobank questionnaires, they may believe their coronary thrombosis to be a heart attack, when in fact in this investigation we assumed by heart attack, the participant was referring to a myocardial infarction, which while similar in its outcome, has different causes.

## Conclusions

Upon conducting MR analysis on European descendants, this investigation has shown some evidence that a higher BMI causes an increased risk of an individual having a heart attack. Therefore, individuals who are overweight should look to reduce their weight and health services should look to producing guidelines that encourage this behaviour.

These findings suggest that parameters should be put in place following consultations with obese and overweight patients that look to induce their weight loss as we now know that it may have a direct causal association with heart attacks. Such parameters could be an automatic appointment with a dietitian or a leaflet on local places to exercise. Should future GWAS re-impute the same data, their questionnaires on heart attacks should contain more specific terminology (i.e. instead of asking whether the patient has had a heart attack, they could ask which *type* of *myocardial infarction* they have had). Then MR analysis could be carried out on BMI’s causal association with each type of myocardial infarction. This could help with reducing wasting the aforementioned parameters on patients who potentially would not benefit from them. Future research could also focus on the action of the proteins the identified SNPs are related to and on understanding their function in the body.

## Data Availability

Not applicable.

## Abbreviations

GIANT: Genetic investigation of anthropometric traits
MR: Mendelian randomisation
SNP: Single nucleotide polymorphism
IVW: Inverse variance weighted estimation
OR: Odds ratio
SD: Standard deviation
CI: Confidence interval
RAPS: Robust adjusted profile score
GWAS: Genome wide association study
LD: Linkage disequilibrium
QC: Quality control
MAF: Minor allele frequency
HWE: Hardy-Weinberg equilibrium
TOMM40: Translocase of outer mitochondrial membrane 40
APOE: Apolipoprotein E
ENGAGE: European network for genetic and genomic epidemiology
IL-6(R): Interleukin 6 (receptor)
CRP: C-reactive protein
